# Phased Traumatic Stress Responses Among Caregivers of Children and Adults Recently Diagnosed with Acute Leukemia: A Grounded Theory Study

**DOI:** 10.3390/curroncol33050255

**Published:** 2026-04-29

**Authors:** Carmine Malfitano, Stephanie M. Nanos, Luigi Grassi, Rosangela Caruso, Gary Rodin

**Affiliations:** 1 Department of Supportive Care, Princess Margaret Cancer Centre, University Health Network, Toronto, ON M5G 2C4, Canada; stephanie.nanos@mail.utoronto.ca (S.M.N.); gary.rodin@uhn.ca (G.R.); 2 Centre for Psychology and Emotional Health, Toronto, ON M5R 2A5, Canada; 3 Institute of Medical Science, University of Toronto, Toronto, ON M5S 1A1, Canada; 4 Child Health Evaluative Sciences, Hospital for Sick Children, Toronto, ON M5G 1X8, Canada; 5 Institute of Psychiatry, Department of Neuroscience and Rehabilitation, University of Ferrara, 44121 Ferrara, Italy; luigi.grassi@unife.it (L.G.);; 6 Integrated Department of Mental Health and Pathological Addictions, Local Health Trust of Ferrara, 44124 Ferrara, Italy

**Keywords:** cancer caregiver, psychosocial care, qualitative, traumatic stress, acute stress disorder, post-traumatic stress disorder, trauma

## Abstract

A diagnosis of acute leukemia (AL) represents a sudden, life-threatening event that places family caregivers (FCs) at high risk for traumatic stress. While traumatic stress symptoms have been documented among FCs later in the cancer trajectory, little is known about how these responses unfold during the immediate peri-diagnostic period, when acute stress disorder (ASD) may emerge, and early intervention could be most impactful. We conducted a qualitative study using a constructivist grounded theory approach to examine early traumatic stress responses among FCs of adults and children with newly diagnosed AL. Semi-structured interviews were conducted with 18 caregivers within the first six months of diagnosis as part of two clinical trials at major cancer centres in Toronto, Canada, and were analyzed iteratively using constant comparative methods. Caregivers described a coherent trajectory of traumatic stress responses across three phases. The anticipatory phase was characterized by prolonged uncertainty, helplessness, and mounting fear during diagnostic investigations. The acute phase, beginning at diagnosis, involved an abrupt shift toward emotional numbing, deliberate avoidance of catastrophic thoughts, and a narrowed focus on immediate tasks, often described as operating on “autopilot.” In the post-acute phase, as patients stabilized and discharge approached, caregivers reported increased emotional access, including grief, anger, and recognition of their own trauma, alongside emerging concerns about long-term caregiving and life disruption. These findings suggest that FCs of individuals with newly diagnosed AL exhibit a phased pattern of traumatic stress responses, marked by an early, adaptive dissociative coping response followed by delayed emotional processing, underscoring the importance of phase-sensitive psychosocial care in oncology.

## Simple Summary

Traumatic stress responses are common among family caregivers of children and adults with cancer, yet little is known about how these reactions unfold over time or how psychosocial care can better support them. In this study, we interviewed family caregivers of individuals recently diagnosed with acute leukemia to better understand their early experiences. Caregivers described a clear pattern of traumatic stress responses across three phases: an anticipatory phase, an acute phase, and a post-acute phase. These findings highlight the need for psychosocial care that is tailored to caregivers’ evolving needs over time and may inform the design of timelier, phase-specific supports to reduce long-term distress.

## 1. Introduction

Traumatic stress symptoms (TSSs) can develop following a traumatic event, which is defined in the DSM-5 as direct or indirect exposure to actual or threatened death or serious injury [1]. These symptoms include intrusive and distressing reminders of the event; avoidance of thoughts, feelings, or external reminders associated with the event; negative alterations in cognition and mood; and heightened arousal or reactivity, such as irritability, hypervigilance, or sleep disturbance. Specific durations and patterns of TSS may meet diagnostic criteria for acute stress disorder (ASD), which is diagnosed within the first month following a traumatic event, or post-traumatic stress disorder (PTSD), which occurs when symptoms persist beyond one month. These conditions are associated with profound functional impairment, increased medical morbidity, and higher mortality [2]. Notably, individuals diagnosed with ASD have a nearly tenfold higher risk of dying by suicide compared to the general population [3,4,5]. Since ASD strongly predicts later PTSD [6], early trajectories of traumatic stress warrant careful clinical attention.

Although TSSs have been well documented in other trauma-exposed populations, their manifestation in oncology remains comparatively understudied. Cancer is not inherently traumatic for all individuals; however, many patients and family members describe the diagnostic and early treatment period as marked by fear, helplessness, and an intense sense of threat [7]. Unlike acute, time-bound traumatic events commonly examined in PTSD research, the cancer trajectory involves repetitive and cumulative exposures, including the moment of diagnosis, invasive treatments, distressing complications, disease progression, and relapse. These stressors are compounded by the perception of cancer as an ongoing, internally located, and often inescapable threat rather than a discrete past event [8]. Recent systematic work underscores that these threats often manifest as clinically significant TSS in a substantial proportion of individuals within weeks of a new diagnosis of advanced cancer [9].

Family caregivers (FCs) play an essential role throughout the cancer experience and are exposed to distinct emotional, relational, and practical stressors. FCs witness their family member’s physical and psychological suffering, navigate complex clinical decisions and medical systems, and provide direct care while simultaneously managing household, occupational, and family responsibilities [10]. Published reviews demonstrate elevated rates of PTSD and TSS among caregivers of individuals with serious medical illnesses [11,12]. Across cancer types, PTSD prevalence in FCs ranges from approximately 3% to 37%, and TSS rates range from 10% to 57% [9]. Despite growing recognition of these vulnerabilities, most caregiver studies in oncology rely on cross-sectional surveys conducted months or years after diagnosis, leaving early post-diagnosis processes poorly understood. This is also the period when ASD is most likely to develop and when preventive psychosocial intervention may be most impactful [2,6].

These limitations are especially consequential in the context of life-threatening cancers, where the diagnostic and initial treatment periods confront families with an abrupt and overwhelming threat [8]. Furthermore, unlike in discrete trauma events, caregivers of individuals with advanced cancer face sustained, unavoidable exposure to the source of threat, making avoidance—the core defensive mechanism in classic trauma models—difficult or impossible [9]. Acute leukemia (AL) is a prototypical example of an acute life-threatening cancer due to its rapid onset and immediate threat to life, requiring the timely initiation of intensive inpatient chemotherapy, leaving families with very little time to prepare emotionally for the crisis that unfolds [13]. The requirement for immediate hospitalization means that patients and their FCs are often present on the unit for extended periods, creating a rare opportunity to study traumatic stress responses unusually close to the diagnostic event [13,14].

FCs of adults with newly diagnosed AL report some of the highest prevalences of PTSD across oncology populations [9,15]. Evidence further indicates that approximately one-third of FCs of adults with AL experience clinically meaningful PTSD symptoms within the first months of illness [16]. Additionally, recent longitudinal observational work demonstrated that up to 61% of parents experience clinically significant TSS within the first 3 months of their child’s new diagnosis of AL [17]. Although early traumatic stress responses among patients in this setting have been described qualitatively [18,19,20], far less is known about how FCs experience the acute phase. In particular, little is known about their early emotional responses immediately preceding and following the moment of diagnosis.

A grounded theory approach is well suited to developing an explanatory, process-oriented account of how FCs respond emotionally and behaviorally to the acute threat posed by a life-threatening diagnosis, how they regulate or constrain emotional experience, and how these processes shift as the illness trajectory unfolds [21]. Such models can inform the development of phase-sensitive psychosocial interventions, particularly as trauma-informed treatments increasingly seek to support families during the first weeks following diagnosis. The present study draws on qualitative interviews conducted within two clinical trials involving FCs of adults and children with newly diagnosed AL, offering an opportunity to explore caregiving experiences across the life course and close to the moment of diagnosis. We conducted a grounded theory analysis to characterize FCs’ early trajectories of traumatic stress, examine how they navigate the immediate emotional disruption that follows diagnosis, and generate hypotheses to guide the development of early psychosocial interventions for families confronting AL. Although caregiving experiences differ across developmental and relational contexts (e.g., parents of pediatric patients versus partners or adult children of adult patients), the present study focuses on identifying shared early traumatic stress processes following diagnosis.

## 2. Materials and Methods

### 2.1. Setting and Recruitment

This analysis draws on qualitative interviews conducted as part of two studies at major centres in Toronto, Canada. The first was a non-randomized feasibility trial of a brief psychotherapeutic intervention for parents of children with newly diagnosed AL (ClinicalTrials.gov NCT05236296; Ontario Cancer Research Ethics Board approval: OCREB #2104). At the time of the interview, parents were receiving the intervention. The second study comprised the qualitative arm of a pre-pilot phase of a multicentre randomized controlled trial evaluating a psychotherapeutic intervention for adults with newly diagnosed AL (ClinicalTrials.gov NCT03836010; University Health Network Research Ethics Board approval: CAPCR/REB #18-5161). Caregiver interviews from both studies were de-identified and analyzed under these existing approvals.

### 2.2. Eligibility and Sampling

Eligible participants were FCs of a child or adult with a new diagnosis of AL. All caregivers interviewed within the first six months of the patient’s diagnosis were eligible for inclusion. Eligible caregivers were approached either in person during inpatient admissions or outpatient appointments or by telephone, depending on availability and clinical context, and invited to participate in a qualitative interview. For the adult sample, all FCs who completed qualitative interviews during the pre-pilot phase were eligible; no additional inclusion or exclusion criteria applied. Recruitment aimed to include every consecutive participant who agreed to participate and continued until thematic saturation was reached, defined as the point at which no new conceptual categories arose in successive interviews. Sampling across caregiver roles aimed to capture variation in caregiving contexts to identify shared experiential processes.

### 2.3. Data Collection

All interviews were conducted by the first author (CM), a clinical social worker with extensive psycho-oncology experience and no pre-existing relationship with FCs. Interviews were semi-structured and invited caregivers to describe their experiences from the earliest symptoms and diagnostic investigations through initial hospitalization and treatment. Follow-up questions probed emotional responses, coping strategies, perceived support, and unmet needs. Interviews with caregivers of adults were conducted primarily in person, with some by telephone, and interviews with caregivers of children were conducted online via Microsoft Teams. Interviews lasted 30–60 min, were audio-recorded, transcribed verbatim, and checked for accuracy. Field notes were written immediately after each interview to capture contextual observations and early analytic insights.

### 2.4. Researcher Reflexivity

The interviewer’s dual role as clinician and researcher required explicit attention to reflexivity. CM’s clinical experience facilitated rapport and empathic engagement but also introduced the risk of interpretive assumptions. To address this, CM recorded reflexive memos after each interview and engaged in regular analytic meetings with the second author (SMN), a PhD candidate with multi-methods experience, who provided an external perspective and challenged emerging interpretations. SMN also wrote memos throughout coding to reflect on how her experience as a caregiver to a parent with cancer shaped her research posture. These practices helped bracket assumptions, enhance theoretical sensitivity, and ensure that analytic decisions were grounded in the data rather than preconceptions.

### 2.5. Data Analysis

Data were analyzed using the iterative stages of constructivist grounded theory [21]. Transcripts were imported into NVivo 10 for data management. Analysis began with initial line-by-line coding to identify significant actions, meanings, and emotional processes in caregivers’ accounts. This was followed by focused coding, where the most theoretically significant and frequently occurring codes were compared across interviews to refine emerging categories. Throughout the analysis, we employed constant comparative methods, examining data within and across participants, between caregiver groups, and across timepoints relative to diagnosis. Memo-writing supported conceptual development and documented analytic decisions, contributing to an audit trail. CM and SMN coded transcripts collaboratively, met regularly to discuss discrepancies, and reconciled differences through discussion. Data collection and analysis proceeded concurrently and continued until sufficient conceptual depth supported the development of a clear theoretical framework. Two trained Master of Social Work students used CM and SMN’s codebook to assess and refine the proposed phases, with final analytic decisions made by the co-authors.

## 3. Results

### 3.1. Sample

Twenty-one FCs participated, including parents of children with AL (*n* = 11), a parent of an adult with AL, partners of adults with AL (*n* = 7), and adult children (≥18 years) caring for their parent with AL (*n* = 2). Most FCs were female (*n* = 16), university-educated (*n* = 16), White (*n* = 17), married (*n* = 17), and had children (*n* = 19) (Table 1).

### 3.2. Overall Trajectory

All transcripts fit the three-phase model, each corresponding to a stage in the patient’s illness trajectory. The first phase, termed the “anticipatory phase,” encompassed the period of diagnostic uncertainty and medical investigations leading up to the confirmation of cancer. The second phase, the “acute phase,” began the moment the FC learned of the diagnosis and extended through the initial weeks of intensive treatment. The final phase, the “post-acute phase,” started as the patient stabilized and prepared for hospital discharge. Below, we present these phases in sequence, with representative quotations from FCs (and field notes where applicable) to illustrate key themes. Ellipses within quotes indicate omitted content, and identifying details have been removed to protect confidentiality. Participant ID numbers follow each quote in parentheses, where IDs beginning with “1” denote caregivers of adults, while those beginning with “0” denote caregivers of children.

### 3.3. Anticipatory Phase: “Sitting on the Fence”

In the anticipatory phase, FCs described struggling with pervasive unknowns about the patient’s condition, coupled with a distressing sense of powerlessness. This phase often involved lengthy waits for tests and results without clear answers. FCs felt “on the fence,” a distressing state caught between hoping for benign explanations and fearing the worst. One spouse explained:

“*For me, the unknown is more stressful than the known. I would rather know exactly what’s going on so I can wrap myself around it than just be sitting on the fence waiting to fall off.*”

(1022)

Many FCs noted how helplessness defined this period as they watched their loved ones suffer intense symptoms they could not alleviate. For example, another spouse recalled the anguish of seeing their partner in decline:

“*It was troubling because there was nothing I could do. She couldn’t eat anything, and she just continued to lose weight and I was thinking: ‘Man, something is happening here.’*”

(1019)

A parent described the combination of exhaustion and heartbreak while awaiting a diagnosis for their child:

“*It was exhausting. Exhausting and frustrating because you couldn’t provide any relief […] Any parent, when one sees their child in that much pain and just getting no answers […] it was exhausting trying to find out, and heartbreaking at the same time.*”

(0048B)

This phase culminated in the moment the diagnosis was finally confirmed. FCs universally recalled that moment as shocking and surreal, often describing it as if “in a bad dream.” Many experienced a brief but intense wave of disbelief. As one participant put it:

“*It’s more of a shock more than anything. Like a bad dream… like somebody gets killed in a car accident.*”

(1002)

Several admitted that their worst fear immediately came to mind: “I thought I was going to lose [patient].” (1022). Others described feeling completely unprepared, as if the “floor dropped out” beneath them. One spouse recounted:

“*Being told that someone I care for so deeply could die… it met me totally unprepared. It really dropped me to my knees. I’m thinking: ‘Holy smokes, this can’t be happening!’*”

(1013)

Despite the shock, many FCs reported a sense of relief—not that “all was well” but that they finally knew what they were dealing with.

### 3.4. Acute Phase: “Dialing It All Back”

The acute phase began with the diagnosis and carried through the initial treatment phase. Immediately after the shock of hearing “cancer” or “leukemia,” most FCs described an abrupt shift: the overwhelming fear of losing their loved one was quickly followed by intentional efforts to “dial it all back” by pushing away negative thoughts and worst-case scenarios in order to cope. One spouse shared how she consciously stopped herself from thinking too far ahead:

“I instantly went to: what would my life be like without [patient] there? You go really far into the future. And then… oh f***… I’ve never thought about this before. I may not have a partner at some point. As soon as you go there… I dialed it all back […] I don’t think that is constructive. Why go to a place like that? He is probably going to be fine.”

(1003)

Similarly, another caregiver avoided dwelling on death, focusing instead on staying “positive”:

“*It’s crossed my mind [that my spouse could die], but I try to remain positive. As far as what might happen or how, I haven’t put a lot of thought into it. I try and stay away from that for now.*”

(1013)

FCs narrowed their focus to the present and the immediate future, which one participant called taking life “one step at a time”:

“*We’re just trying to think about one step at a time. We can’t control anything in terms of the illness, so we’re trying not to look too far ahead. Just focus on the next few weeks that we’re here [in the hospital], and deal with home once we’re there. Just trying to get through each week as it comes.*”

(1003)

FCs concentrated on concrete, actionable tasks (e.g., arranging care, learning medical information, monitoring day-by-day progress) rather than contemplating long-term uncertainties. As one caregiver explained:

“*My anxiety levels shot right through the top at first, and then I backstepped myself. I said: ‘Okay, stop thinking that way. Get in control, get knowledge.’*”

(1022)

Many FCs also reported feeling a temporary emotional numbness, as if operating on “autopilot.” They described automatically shifting into a pragmatic “caregiver mode”, focusing on what needed to be done rather than their own feelings. In the words of three caregivers:

“*I would have expected myself to just fall apart… I didn’t. I just felt almost like… numb. Numb is probably a good description. Almost like on autopilot.*”

(1001)

“*You have those initial very like shock and super sad feelings [but] it’s almost like this switch turns on and you just kind of go, OK, well, I have to step out of this and—and what can we do next? […] I had to kind of push my feelings back so that I didn’t just break down all the time.*”

(0047B)

“*I go into strong mode. I am there to take care of all [his] needs. I go into caregiver mode… I just go into autopilot.*”

(1003)

Field notes corroborated this apparent lack of outward distress in the early acute phase: most caregivers interviewed just days after admission appeared surprisingly composed compared to FCs interviewed later. This pragmatic, emotionally distanced coping style generally persisted throughout the early treatment phase. However, several caregivers noted that this state could be suddenly disrupted by worrisome changes in the patient’s health. Witnessing intense pain or frightening complications (such as relentless vomiting, high fevers, or other side effects of chemotherapy) was described as the most distressing aspect of caregiving. In those moments, fear and anguish resurfaced. One spouse recounted an episode during the second week of induction chemotherapy:

“*It was exceptionally difficult… he was very ill. I found it overwhelming to be here and watch him vomiting, have them tell us he’s fevered. And suddenly that move from, you know, sad, shocked, numb to terrified.*”

(1001)

### 3.5. Post-Acute Phase: “Living with Trauma”

As patients’ conditions stabilized and the focus shifted toward discharge, FCs entered what we termed the post-acute phase. During periods when their loved one’s health appeared to improve, FCs described feeling as though they could “take a breath”, with such moments occurring more frequently as time from diagnosis increased. Observing signs of recovery, such as the patient regaining strength, fostered a sense that “things might be okay.” One spouse noted:

“*[Compared to before,] I am a little more calm this week. I guess it’s just a realization of what’s going on, what’s happening. You can see the changes, she looks good today. So that makes you feel better.*”

(1022)

Another spouse described how a simple act by her recovering husband eased her anxiety:

“*In the last two nights when I left, I realized the reason I am feeling more under control is because he said, ‘Oh, I could walk you to the elevator.’ So I left a man of strength.*”

(1001)

However, “living with” the traumatic experience seemed to begin in this phase. Several weeks removed from the diagnosis, FCs described a delayed onset of emotional processing. With the immediate crisis past, they began to feel and reflect on the depth of what had happened:

“*While you were in the thick of it, you don’t really think of [yourself] because you’re just—your priority is just to make sure that your child’s OK. But now that she’s on the other side, you’re kind of like, “OK, I need to get some sense of relief here.*”

(0048b)

We observed that caregivers interviewed near discharge were more openly emotional than those interviewed earlier, with transcripts and field notes more frequently indicating tears or expressions of grief and anger. One spouse, interviewed three weeks after admission (one week before her husband’s discharge), exemplified this delayed emotional release. When asked about the future, she became tearful and said:

“*Well, it was tough. It was tough (crying). And I think part of it is anger built in. There is anger, and anger comes with grief, right? […] As much as this is happening to him, I also realized that this is happening to me and the life we had together (continues crying).*”

(1001)

In this statement, she acknowledges her own suffering, recognizing that she, too, has been traumatized by the illness. FCs in the post-acute phase commonly noted that only with time and a degree of stability could they afford to widen the scope of their thoughts and feelings. In other words, being further removed from the initial shock created space for reflection on what they had endured, as illustrated by one mother:

“*When we got to the end [of the first month] knowing that she was in remission […] gave me a sense of like, okay, she’s doing better now. How do I really feel about everything we’ve been going through?*”

(0047b)

As discharge approached, this reflective space was accompanied by the emergence of new worries:

“*I guess compared to [the beginning of the diagnosis] I definitely spend a lot more time worrying, for a lack of better words, [about] what’s gonna come next.*”

(0047b)

“*What’s going to happen when (partner) is released. How are we going to handle it?*”

(1013)

FCs expressed concerns about the lengthy treatment course, leaving the perceived safety and support of the hospital and their healthcare team, potential exposure to infections at home, home modifications for the patient’s weakened state, and other practical limitations the illness imposed:

“*I know it is important for our mental health to not constantly worry about the future [but] I am overwhelmed by the length of what this journey is going to be.*”

(1001)

“*When he goes home […] I do have anxiety about “oh there is no nurses watching for his fever, it is me”. There is nobody making the call if we are going to the emergency or not. So, I am stressed about that.*”

(1001)

In essence, once the immediate crisis subsided, FCs started to grapple with “what now?”—coming to terms with a “new normal” after a traumatic medical event.

## 4. Discussion

The diagnosis, progression, or recurrence of advanced cancer can be a traumatic life event with profound psychological and physical consequences for adult and pediatric patients and their family caregivers. In particular, the fear of death, the sense of inescapable threat, invasive medical procedures, and the presence of distressing symptoms can contribute to the emergence of TSS in both populations [7,8]. Prior research has demonstrated significant prevalence rates of ASD and PTSD in cancer patients and their FCs later in the illness trajectory [22]. However, the moment of diagnosis and the critical weeks that follow have received minimal research attention, and virtually none focused on family caregivers [9]. This gap has hindered the development of specialized early interventions to alleviate TSS in oncology settings [12].

The present study is among the first to characterize the immediate post-diagnosis experience of FCs of AL patients, a context that exemplifies the abrupt onset of a life-threatening cancer [13]. Our qualitative findings reveal that FCs’ traumatic stress responses closely parallel those previously described in patients with AL [19]. This underscores the unique psychological burden of witnessing a family member suffer through a sudden medical crisis and supports dyadic models of coping, which posit that patients and their close others function as a “system of mutual influence,” each affecting the other’s emotional responses [23,24,25,26,27]. FCs also described a marked shift during the discharge transition, with emerging concerns about infection risk, caregiving responsibilities, and the loss of continuous hospital monitoring, echoing transition-related challenges previously reported by patients with AL [20]. By focusing on FCs in the earliest phase of illness, we also contribute to the broader trauma literature by describing the very acute stage of adaptation in a cancer context.

A clear pattern that emerged from the interviews was FCs’ immediate protective response to the painful experience of diagnosis. Importantly, this response did not occur in an emotional vacuum but followed a period of prolonged uncertainty, helplessness, and anticipatory fear during the diagnostic workup. In all cases, the overwhelming surge of fear upon hearing the diagnosis was followed almost reflexively by a protective psychological mechanism: FCs intentionally avoided “dangerous” thoughts about death or loss, engaged themselves in concrete tasks or fact-finding, and clung to hopeful possibilities (e.g., the prospect of a cure). This response can be interpreted as an acute coping strategy that serves an adaptive function, essentially allowing FCs to keep functioning and supporting the patient in the face of potential emotional flooding. Only later, as the patient stabilized, did previously suppressed emotions begin to emerge, when many FCs could reflect on and articulate feelings of grief, anger, and personal loss. This signals a gradual softening of initial emotional numbness and growing access to underlying distress.

These findings carry important clinical implications. They suggest that FCs’ very early response to a life-threatening diagnosis might be self-protective and should be respected as such. In fact, established early post-trauma guidance emphasizes supportive, stabilizing responses and cautions against interventions aimed at evoking emotions delivered immediately after trauma exposure [28]. During this initial crisis phase, psychosocial interventions might better focus on building a strong therapeutic alliance and normalizing the caregiver’s experience of shock and numbness, thereby providing validation and basic emotional support. Simple, present-oriented tools such as psychoeducation about common early trauma reactions, helping the caregiver prioritize immediate tasks, and teaching basic techniques to regulate acute anxiety could be most appropriate at this stage.

As the caregiver progresses beyond the initial phase, a more flexible, stage-based therapeutic approach is warranted. For example, in the post-acute phase, when FCs start to express grief or anger, clinicians can provide empathic understanding and validation, gently encourage those discussions, and assist in meaning-making. Therefore, the ability of a therapist or support provider to toggle between supportive and exploratory interventions requires careful attunement to the caregiver’s current state. Overall, a phase-informed approach to caregiver support may maximize effectiveness: early stabilization and validation, followed by later opportunities for deeper emotional work.

From a research perspective, these insights open several avenues. Future studies should examine whether the phased pattern of traumatic stress responses we identified in AL caregivers generalizes to caregivers in other cancers or acute medical crises. It would be valuable to investigate, for instance, if caregivers of patients with other sudden-onset, life-threatening conditions show a similar trajectory of initial dissociative coping followed by delayed emotional processing. Additionally, our sample included FCs who were receiving a psychosocial intervention (in the pediatric caregiver group) as well as those who were not (adult caregiver group); future analyses could explore how such interventions might modulate or shorten the phases of distress [29,30]. Finally, intervention research should consider timing and tailoring: designing caregiver-support programs that deliver the right type of help at the right phase (e.g., practical support and crisis management early on, transitioning to therapy techniques targeting PTSD prevention or emotional processing later).

## 5. Conclusions

This qualitative study explored the experiences of family caregivers in the weeks following a new diagnosis of acute leukemia. Using a grounded theory approach, we identified three sequential phases in the caregiver experience: an anticipatory phase of mounting distress and uncertainty leading up to the diagnosis, an acute phase marked by shock and a dissociative coping response that blunted overwhelming emotions, and a post-acute phase where deeper feelings resurfaced as the patient stabilized. FCs’ distress appeared to peak at the moment of diagnosis and then decline sharply soon thereafter, coinciding with their reported emotional numbness and narrow focus on immediate tasks. This initial adaptive response was characterized by a conscious emphasis on positive outcomes, present-oriented information, and actionable tasks, which may serve to protect caregivers from debilitating anxiety in the short term. As time progressed, caregivers regained access to their underlying emotional states and began processing the impact of the illness on their lives.

These findings suggest that early traumatic stress responses in caregivers have an adaptive component that buffers them during the crisis. Future psychotherapeutic interventions for families confronted with acute cancer diagnoses should take into account the protective nature of this early response, providing support and stabilization initially and then gradually facilitating emotional processing in later stages. By aligning support strategies with the caregiver’s phase of adaptation, we may improve psychosocial outcomes for families navigating the trauma of a life-threatening cancer diagnosis.

## 6. Limitations

Several limitations of this study should be acknowledged. First, our findings are based on a specific population: FCs of patients who had recently been diagnosed with acute leukemia. This narrow focus may limit generalizability to other illness contexts or traumatic events occurring at different points in the cancer trajectory, such as relapse or end-of-life care. Second, participants were recruited from large urban cancer centres in a major Canadian city; caregivers in rural or resource-limited settings might have different experiences that were not captured here.

There is also potential selection bias in our sample: we included only patients who identified a family caregiver and only those caregivers who consented to be interviewed. Caregivers facing extreme distress or lacking sufficient support may have been less likely to participate, which could have influenced the findings. In addition, we did not systematically record the number of caregivers who declined participation or their reasons for refusal, further limiting our ability to assess selection bias.

Finally, participants were recruited within the context of clinical trials, and a subset of them (parents of pediatric patients) were receiving a psychosocial intervention at the time of the interview, which may have influenced how they reflected on earlier experiences. However, caregivers of adult patients were not exposed to an intervention, and similar patterns were observed across both groups. Moreover, many of the processes described appeared to reflect immediate responses to the diagnosis rather than intervention-related effects.

Additionally, as a qualitative grounded theory study, our analysis reflects the research team’s interpretations of the data. While we employed rigorous procedures, we did not include formal member-checking with participants. The absence of external validation steps means there is a possibility that researcher bias influenced the themes, despite reflexive practices. Finally, roughly half of our caregiver participants (the parents in the pediatric group) were in the process of receiving a psychosocial intervention during the study. While this context provided a unique window into early experiences, it might also have affected how those caregivers coped or what they emphasized in interviews, and we did not formally compare the intervention versus non-intervention subgroups.

## Figures and Tables

**Table 1:** Demographics of family caregivers.

Demographics		FCs of Adults (*n* = 10)	FCs of Children (*n* = 11)
Age	Average	60.2	41.09
	Range	44–71	33–64
Caregiver Type	Parent	1	11
	Son/Daughter	2	0
	Partner	7	0
Sex	Male	4	1
	Female	6	10
Marital Status	Married/Common Law	7	10
	Separated/Divorced	2	1
	Single	1	0
Religion	N/A	6	3
	Catholic	2	4
	Christian	0	4
	Other	2	0
Ethnicity	White	8	9
	Other	2	2
Children	Yes	8	11
	No	2	0
Education	High School	3	2
	College/University	7	9

## Data Availability

Data are available upon request.

## Abbreviations

The following abbreviations are used in this manuscript:

TSS	Traumatic Stress Symptoms
ASD	Acute Stress Disorder
PTSD	Post-Traumatic Stress Disorder
FC	Family Caregiver
